# Multi-Shell Hybrid Diffusion Imaging (HYDI) at 7 Tesla in TgF344-AD Transgenic Alzheimer Rats

**DOI:** 10.1371/journal.pone.0145205

**Published:** 2015-12-18

**Authors:** Madelaine Daianu, Russell E. Jacobs, Tara M. Weitz, Terrence C. Town, Paul M. Thompson

**Affiliations:** 1 Imaging Genetics Center, Mark & Mary Stevens Neuroimaging & Informatics Institute, University of Southern California, Marina del Rey, CA, United States of America; 2 Department of Neurology, UCLA School of Medicine, Los Angeles, CA, United States of America; 3 Division of Biology and Biological Engineering, Beckman Institute, California Institute of Technology, Pasadena, CA, United States of America; 4 Department of Physiology & Biophysics, Zilkha Neurogenetic Institute, University of Southern California, Los Angeles, CA, United States of America; 5 Departments of Neurology, Psychiatry, Radiology, Engineering, Pediatrics, and Ophthalmology, University of Southern California, Los Angeles, CA, United States of America; University of North Carolina, UNITED STATES

## Abstract

Diffusion weighted imaging (DWI) is widely used to study microstructural characteristics of the brain. Diffusion tensor imaging (DTI) and high-angular resolution imaging (HARDI) are frequently used in radiology and neuroscience research but can be limited in describing the signal behavior in composite nerve fiber structures. Here, we developed and assessed the benefit of a comprehensive diffusion encoding scheme, known as hybrid diffusion imaging (HYDI), composed of 300 DWI volumes acquired at 7-Tesla with diffusion weightings at *b* = 1000, 3000, 4000, 8000 and 12000 s/mm^2^ and applied it in transgenic Alzheimer rats (line TgF344-AD) that model the full clinico-pathological spectrum of the human disease. We studied and visualized the effects of the multiple concentric “shells” when computing three distinct anisotropy maps–fractional anisotropy (FA), generalized fractional anisotropy (GFA) and normalized quantitative anisotropy (NQA). We tested the added value of the multi-shell *q*-space sampling scheme, when reconstructing neural pathways using mathematical frameworks from DTI and *q*-ball imaging (QBI). We show a range of properties of HYDI, including lower apparent anisotropy when using high *b*-value shells in DTI-based reconstructions, and increases in apparent anisotropy in QBI-based reconstructions. Regardless of the reconstruction scheme, HYDI improves FA-, GFA- and NQA-aided tractography. HYDI may be valuable in human connectome projects and clinical research, as well as magnetic resonance research in experimental animals.

## Introduction

Diffusion-weighted imaging (DWI) is a powerful and widely used tool to study water diffusion in the brain. This variant of magnetic resonance imaging was developed to be sensitive to the Brownian motion of water molecules in the living brain; as diffusion is directionally constrained and reflects tissue microstructure, DWI can yield insights into the microstructural wiring of the nervous system. The random motion of molecules in the white matter is hindered by organized bundles of neural tissue, and DWI signals can reveal the architecture of the neuronal connections that make up the human connectome. Many recent developments focus on increasingly detailed descriptors of local diffusion–aiming to achieve more accurate connectivity analyses and better understanding of the underlying neuroimaging signal.

Statistical measures of local diffusion can be estimated from a minimum of six independent diffusion-sensitized images, and one non-diffusion weighted image (also known as the *b*_*0*_ image). These images are used to model diffusion anisotropy in diffusion tensor imaging (DTI)–the first proposed approach designed to estimate a 3x3 diffusion tensor [1], or the covariance matrix of a 3-dimensional Gaussian distribution. This model is a successful initial approach that has been widely applied in clinical research. However, DTI often fails to resolve complex fiber orientations in the white matter bundles, especially in regions where fibers mix and cross and where diffusion within an imaged voxel cannot be approximated as Gaussian. In addition, with DTI it is hard to model partial volume effects–where white matter, gray matter and cerebrospinal fluid may all contribute to diffusion in the same voxel [2] [3]. In these cases, the DTI model will fail to reconstruct neuronal structures [3] [4] and more complex mathematical frameworks are needed, as well as more sophisticated acquisition protocols.

To overcome some limitations of DTI, a broad spectrum of acquisition and reconstruction methods have been developed [5]. For example, high angular resolution diffusion imaging (HARDI) acquires a large number of angular measures of diffusion in a “single-shell” scheme (*i*.*e*., using a fixed diffusion weighting, or *b*-value). Other methods, such as diffusion spectrum imaging (DSI) [4] [6] [7] [8], use dense Cartesian sampling [9] at a large set of possible angles and multiple *b*-values to resolve diffusion microstructures with a model-free approach. A large number of measurements are needed to encode the *q*-space at each voxel [10], so DSI can be time consuming limiting its application in clinical context. An alternative approach, which we use here, employs a multi-shell scheme to sample diffusivity on multiple concentric spheres in *q*-space, each with a fixed *b*-value [10]. This method is referred to as multi-shell HARDI, or *hybrid diffusion imaging* (HYDI). Although we and others have used this method in humans [10] [11] [12] [13] and animal models [14], this paper is the first report of HYDI with 300 diffusion volumes in transgenic Alzheimer rats, where longer scan times can resolve even finer structure. This allows us to understand how the specifics of image acquisition contribute to the observed microstructural measures, and which aspects of the imaging protocol are most beneficial in terms of the kinds of information acquired.

In HYDI, the diffusion attenuation is sampled on several *q*-sampling spheres, rather than on a Cartesian lattice as in DSI. The multiple sampling spheres in *q*-space offer a trade-off between DSI and classical HARDI techniques in *q*-ball imaging (QBI) [15]. This way, HYDI can provide data needed for a range of diffusion reconstruction methods–such as DTI, QBI, and DSI–without the requirement for rectilinear sampling of *q*, which can be more time-efficient. Furthermore, the lower *b*-value shells offer high signal-to-noise ratio (SNR), whereas the high *b*-value shells offer high angular contrast-to-noise ratio (CNR) [11] [16] [17]; together, they should offer better characterization of complex tissue organization.

In this study, we utilized the TgF344-AD transgenic rat model of Alzheimer’s disease (AD) that over-expresses “Swedish” mutant human amyloid precursor protein and Δ exon 9 mutant human presenilin-1, and progressively develops cognitive impairment and the full spectrum of AD-like pathological features [18]. We scanned three of the TgF344-AD engineered rats with high-field HYDI and acquired 300 diffusion volumes at 5 distinct *q*-sampling shells: *b* = 1000, 3000, 4000, 8000 and 12000 s/mm^2^, which was the largest range of *b*-values that our hardware would deliver. This novel 5-shell acquisition protocol in experimental Alzheimer rats makes this study particularly unique. Furthermore, we were especially interested in understanding the changes brought by each added shell on (1) various anisotropy maps that are widely used in radiologic and neuroscience research (fractional anisotropy, generalized fractional anisotropy and normalized quantitative anisotropy) and (2) the ability to resolve major pathways within the white matter. We combined the *q*-sampling shells starting from the low *b*-values, where 1-shell included only the *b* = 1000 s/mm^2^, 2-shell included *b* = 1000 and 3000 s/mm^2^ and so on, while 5-shell included all 5 *q*-sampling shells. We hypothesized that the latter would lead to highly detailed anisotropy maps and coherent fiber orientations in the white matter, consistent with known anatomical fiber architecture. We also hypothesized that the 1-shell reconstruction would perform worst in resolving major fiber orientations. Finally, we provide a summary of anisotropy measures, how HYDI schemes affect them, and a demonstration of FA-, NQA- and GFA-aided tractography based on HYDI.

## Methods

### Animals

Three transgenic Alzheimer rats (line TgF344-AD) were generated on a Fischer 344 background by co-injecting rat pronuclei with two human genes driven by the mouse prion promoter: “Swedish” mutant human APP (APP_sw_) and Δ exon 9 mutant human presenilin-1 (PS1ΔE9) [18]. Transgene integration was confirmed by genotyping and expression levels were evaluated by Western blot of brain homogenates. All experiments were conducted with protocols approved by the Institutional Animal Care and Use Committee (IACUC). The protocol called ‘Peripheral TGF-beta Pathway Inhibitor Therapy in Alzheimer's Rats’ was approved by the University of Southern California IACUC (Protocol Number: 20044). TgF344-AD rats were housed at the University of Southern California, Zilkha Neurogenetic Institute animal facility. Rats were maintained on normal lab chow and generally housed two per cage, in order to allow socialization. Nesting material was provided to all rats, and environmental enrichment in the form of plastic vertical barriers or tubes was added to all cages. Additionally, extraneous noise that may induce stress was minimized by keeping doors closed to housing rooms. A cage cleaning protocol was adopted that balanced hygiene with the need to retain some odor cues (e.g. scent-marked nesting material) to avoid stress and aggression. Finally, gentle and frequent handling of rats early in life was ensured. Before scanning, we anesthetized the animals with isoflurane, and then performed euthanasia. All efforts were made to minimize suffering.

### Image Acquisition and Processing

We scanned the three rats *ex vivo* at 10, 15 and 24 months with a 7 Tesla Bruker BioSpin MRI scanner at California Institute of Technology. After the three rats were sacrificed at the aforementioned ages, fixed brains (intact within the skull) were soaked in a gadolinium contrast agent (5mM ProHance) for 4 days prior to imaging to decrease the overall T1 of the tissue [19]. To ensure no leakage and that the signal would not change during acquisition, the samples were immersed in galden (perfluoropolyether with same magnetic susceptibility as water). During acquisition, the temperature was monitored via a fiber optic temperature sensor near the sample (and was 20°C for the whole scan).

We acquired high-resolution fast low angle magnetic shot (FLASH) anatomical images with a mix of T1 and T2 weighting (375x224x160 matrix; voxel size: 0.08x0.08x0.08 mm^3^; TR/TE = 50 ms/9ms; pulse angle = 50°). Using a 3D 8-segment spin echo EPI sequence with 1 average, we acquired 300 DWI volumes (133x233x60 matrix; voxel size: 0.15x0.15x0.25 mm^3^; TE = 34 ms; TR = 500 ms; δ = 11 ms; Δ = 16 ms), yielding a 20-hour scan time. Specifically, 60 DWI volumes were acquired for each of 5 *q*-sampling shells, *b* = 1000, 3000, 4000, 8000 and 12000 s/mm^2^, with the same angular sampling, and 5 T2-weighted volumes with no diffusion sensitization (*b*_*0*_ images). The relatively long δ and Δ values were required to achieve the largest *b*-values within the duty cycle constraints of our gradient coils. In addition, it is important to note that although long scan time precludes *in vivo* imaging, it is not an issue for fixed samples [20].

During preprocessing, extra-cerebral tissue was removed using the “skull-stripping” Brain Extraction Tool from BrainSuite (http://brainsuite.org/) for both the anatomical images and the DWIs. We corrected for eddy current distortions using the “eddy correct FSL” tool (www.fmrib.ox.ac.uk/fsl) for which a gradient table was calculated to account for the distortions. DWIs were up-sampled to the resolution of the anatomical images (with isotropic voxels) using FSL’s *flirt* function with 9 degrees of freedom; the gradient direction tables were rotated accordingly after each linear registration.

We calculated the signal-to-noise (SNR) ratio of each *q*-sampling shell. To do this, we computed the average diffusion signal in the white matter structure of each rat and estimated the noise from the mean standard deviation of all 5 *b* = 0 s/mm^2^ images.

### Computing Diffusion Anisotropy Maps

First, we reconstructed standard diffusion tensors using the diffusion tensor imaging (DTI) model to obtain fractional anisotropy (FA) maps in 1-shell (*b* = 1000 s/mm^2^), and multi-shell reconstructions: 2-shell (*b* = 1000 and 3000 s/mm^2^), 3-shell (*b* = 1000, 3000 and 4000 s/mm^2^), 4-shell (*b* = 1000, 3000, 4000 and 8000 s/mm^2^) and finally, 5-shell reconstruction using all data from all 5 shells. FA is one of the most cited DWI metrics in studies of neurodegeneration and is a function of (1) the first eigenvalue of the diffusion tensor, *λ*_1_, also known as axial diffusivity (AX) describing the direction of maximal ‘apparent’ diffusion, and (2) the second and third eigenvalues, *λ*_2_ and *λ*_3_, embedded in the plane orthogonal to the main diffusion [1]:

$$FA=\sqrt{\frac{3}{2}\frac{{\left(\lambda_{1}-MD\right)}^{2}+{\left(\lambda_{2}-MD\right)}^{2}+{\left(\lambda_{3}-MD\right)}^{2})}{\lambda_{1}{}^{2}+\lambda_{2}{}^{2}+\lambda_{3}{}^{2}}}$$ (1)

The average of all eigenvalues describing the diffusion ellipsoid, $MD=\frac{\lambda_{1}+\lambda_{2}+\lambda_{3}}{3}$, is referred to as the mean diffusivity (MD), while the average of the second and third eigenvectors define radial diffusivity (RD), $RD=\frac{\lambda_{2}+\lambda_{3}}{2}$ [1]. These metrics have been shown to be valuable in the tracking of AD as well as normal and abnormal development [21] [22] [23] [24] [25] [26].

Furthermore, we computed orientation distribution functions (ODFs) using *q*-ball imaging (QBI) [27] with a spherical harmonic order of 8 to ensure high angular resolution, and a recommended regularization parameter of 0.006. QBI can resolve complex intravoxel white matter structure and was used here to compute generalized fractional anisotropy (GFA) [4] and normalized quantitative anisotropy (NQA) maps in DSI Studio (http://dsi-studio.labsolver.org/). GFA is directly obtained from the ODF function and is an analogue for QBI of the FA in DTI. GFA is a metric of variation of the ODF and is defined at each voxel:

$$GFA=\sqrt{\frac{n\sum_{i=1}^{n}{\left(\psi (u_{i})-\langle \psi \rangle \right)}^{2}}{(n-1)\sum_{i=1}^{n}\psi {\left(u_{i}\right)}^{2}}}$$ (2)

Here, *n* is the number of discretized ODF profiles; the diffusion ODF, *ψ*(*u*), is computed as the radial projection of the diffusion function [4]:

$$\psi (u)=\frac{1}{Z}\int_{0}^{\infty }P(ru)dr$$ (3)

where *u* is the fiber orientation, *P* is the conditional diffusion probability function, *r* is the relative spin displacement and *Z* is a dimensionless normalization constant [4]; furthermore, $\langle \psi \rangle =\frac{1}{n}\sum_{i=1}^{n}\psi (u_{i})$ is the mean ODF. For an in-depth description of the GFA metric extracted from QBI, please see [4].

Unlike GFA, quantitative anisotropy (QA) is computed from the peak orientation of each ODF and is defined for each fiber orientation [3]:

$$QA(u)=Z_{0}(\psi (u)-I\psi )$$ (4)

Here, *Iψ* computes the isotropic component of the ODF and *Z*_0_ is a scaling factor used to make the maximum of all *Iψ* equal to 1. Furthermore, to make them comparable across subjects, we normalized the maximum QA values to 1 (NQA).

From the 5-shell anisotropy maps computed across all rats we created a minimum deformation template (MDT) and elastically registered all single and multi-shell anisotropy maps to the MDT to align all images into the same space. We also applied the resulting 3D deformation fields to all 300 diffusion volumes [28]. Then, we applied a Gaussian kernel of 3 to all anisotropy maps and ran a voxel-wise linear regression in all rats across the single and all multi-shell maps separately (FA first, then GFA and NQA) to test for changes in anisotropy with each added *b*-value shell. Gaussian smoothing is frequently used in voxel-based analyses to reduce the adverse effects of small residual image misalignments and to improve the SNR [29]. We controlled for age and corrected for multiple comparisons testing using the False Discovery Rate (FDR) at *q*<0.05 [30]. The area of white matter studied in the voxel-wise analyses is delineated in the corresponding figure.

### Angular Deviation Computation

We computed the largest local maximum in the ODFs within each voxel in the corpus callosum and determined the minimum angular deviation, or the inner angle between the fiber orientations corresponding to the local maxima, between each target image (1-shell through 4-shell) and the 5-shell image:

$$\theta =\min\left|\cos^{-1}\left|\sum u_{\max,1}\cdot u_{\max,2}\right|\right|$$ (5)

Here, *u*_max,1_ and *u*_max,2_ are the local maxima peaks within a voxel in the target images and the 5-shell image. We studied the corpus callosum, due to its homogenous fiber distribution. This helped limit biological variance in the angular deviation calculation by avoiding heterogeneous regions. Furthermore, fiber reconstruction in the selected area of the corpus callosum is feasible with both tensor- and ODF-based models, unlike regions with crossing fibers.

To test if the addition of each *q*-sampling shell changed the angular deviation between the target images and the 5-shell images in all rats, we ran a linear regression across the mean angular error in the corpus callosum (by coding each target image with 1 through 5, with “1” depicting the 1-shell images and “5” the 5-shell images). For verification purposes, in a separate analysis we also covaried for age to ensure that our results are not driven by age effects.

### Tractography

We performed FA-, GFA- and NQA-aided tractography focusing on the fibers in the corpus callosum to fully understand the effects of fiber deviation from the previous section. We ran deterministic streamline tractography based on the DTI and QBI reconstructions across the 1-shell through 5-shell images, varying the fiber termination parameters. First, we used FA as a fiber-stopping criterion at thresholds 0.25 for the 1-shell images and 0.20 for the multi-shell reconstructions, as learned from our voxel-wise analyses with anisotropy maps. Then, the GFA fiber tracking stopping criterion was set to 0.02 for the 1-shell images and 0.03 for the multi-shell images. Finally, for NQA, we used stopping threshold of 0.25 for the 1-shell images and 0.30 for the multi-shell images. We traced 300 fibers in the selected area of the corpus callosum at a maximum fiber turning angle of 60°, as suggested by recent studies [31] to optimize specificity and sensitivity in DTI and QBI.

### Diffusion Anisotropy in the Gray Matter

Our co-authors have previously shown that the same TgF344-AD rat model indicated progressive neurodegeneration of the Alzheimer type in these animals. The rats showed consistent and extensive neuronal loss on electron microscopy in the hippocampal region and cortical areas [18]. To evaluate differences in diffusion anisotropy in these AD relevant nuclei, we computed mean FA, NQA and GFA in the hippocampus of all rats at 1-shell and all multi-shell reconstructions. We tested if apparent anisotropy in the hippocampus changed with the increasing number of shells by running a linear regression on each mean anisotropy value, as described above (by coding each mean from the target images with 1 through 5, with “1” depicting the mean computed from the 1-shell images and “5” the 5-shell images). As mentioned above, we also ran a separate analysis by adding age as a covariate.

## Results

### Changes in Apparent Anisotropy with Increasing Number of *q*-Sampling Shells

**Fig 1:**
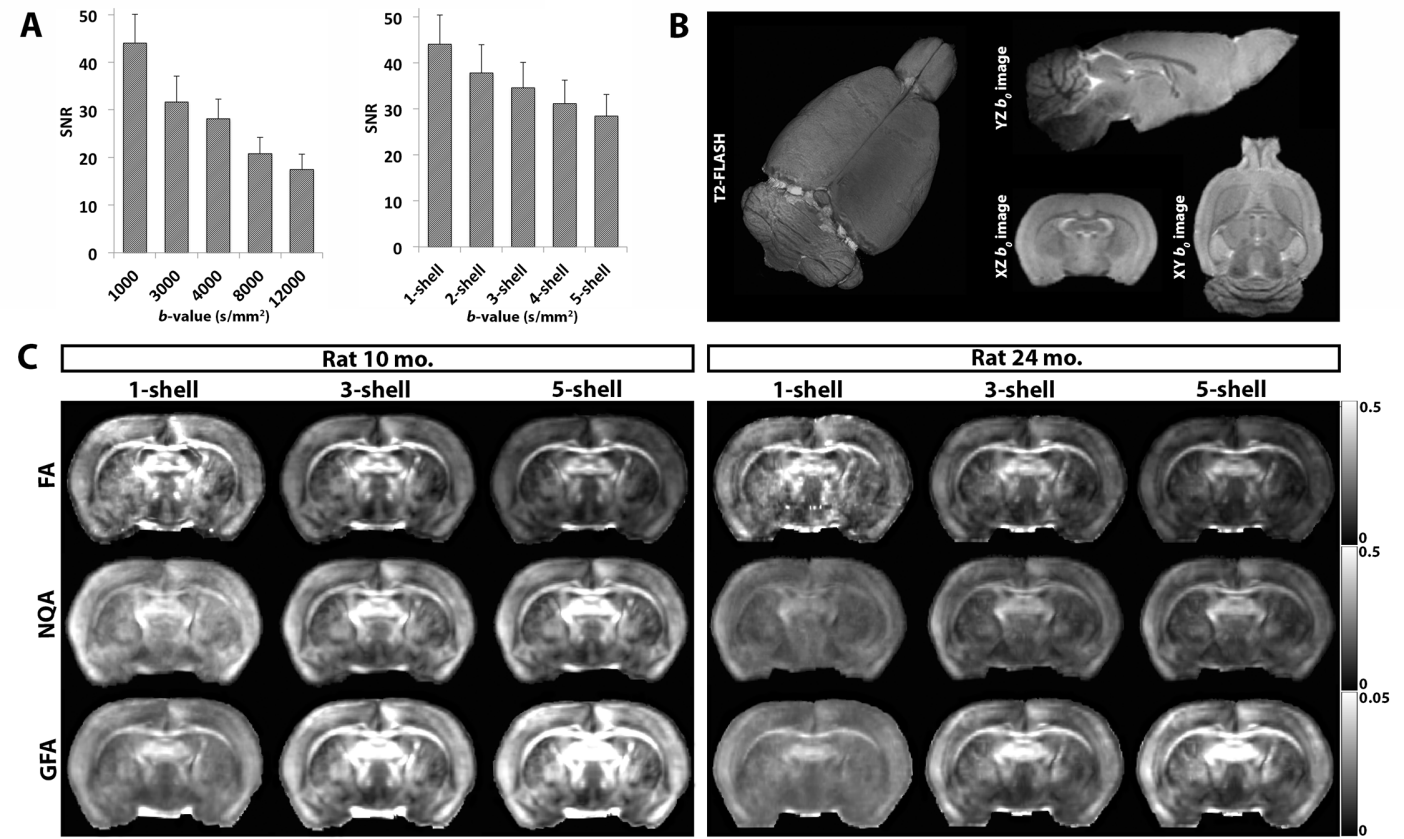
Signal-to-noise ratio (SNR) obtained from the single- and multi-shell diffusion signal and illustration of anisotropy maps. **A.** Average SNR computed from the diffusion signal in *b*-value shells 1000, 3000, 4000, 8000 and 12000 s/mm^2^, and from the 2-, 3-, 4- and 5-shell images. Error bars indicate standard error. **B.** 3D illustration of a T2 anatomical FLASH image and cross sectional illustration of the axial, coronal and sagittal view of the *b*_*0*_ image. **C**. FA, GFA and NQA anisotropy maps in transgenic Alzheimer rats scanned at 10 and 24 months. Note the visibly improved contrast-to-noise ratio in the multi-shell anisotropy maps.

FA, GFA and NQA metrics can be affected by the diffusion sampling scheme and the *b*-value [32] (**Fig 1**), which sensitizes the signal to different aspects of water diffusion. Here, we investigated the changes that take place across single- and multi-shell reconstructions to understand the outcomes from tensor-, ODF-based and fiber density based anisotropy at various *b*-values, and to determine the optimal anisotropy values for fiber termination parameters when running tractography. To start, in **Fig 1A**, we show the expected decrease in SNR with increasing *b*-values and how this affects the CNR in the anisotropy maps (**Fig 1C**).

**Fig 2:**
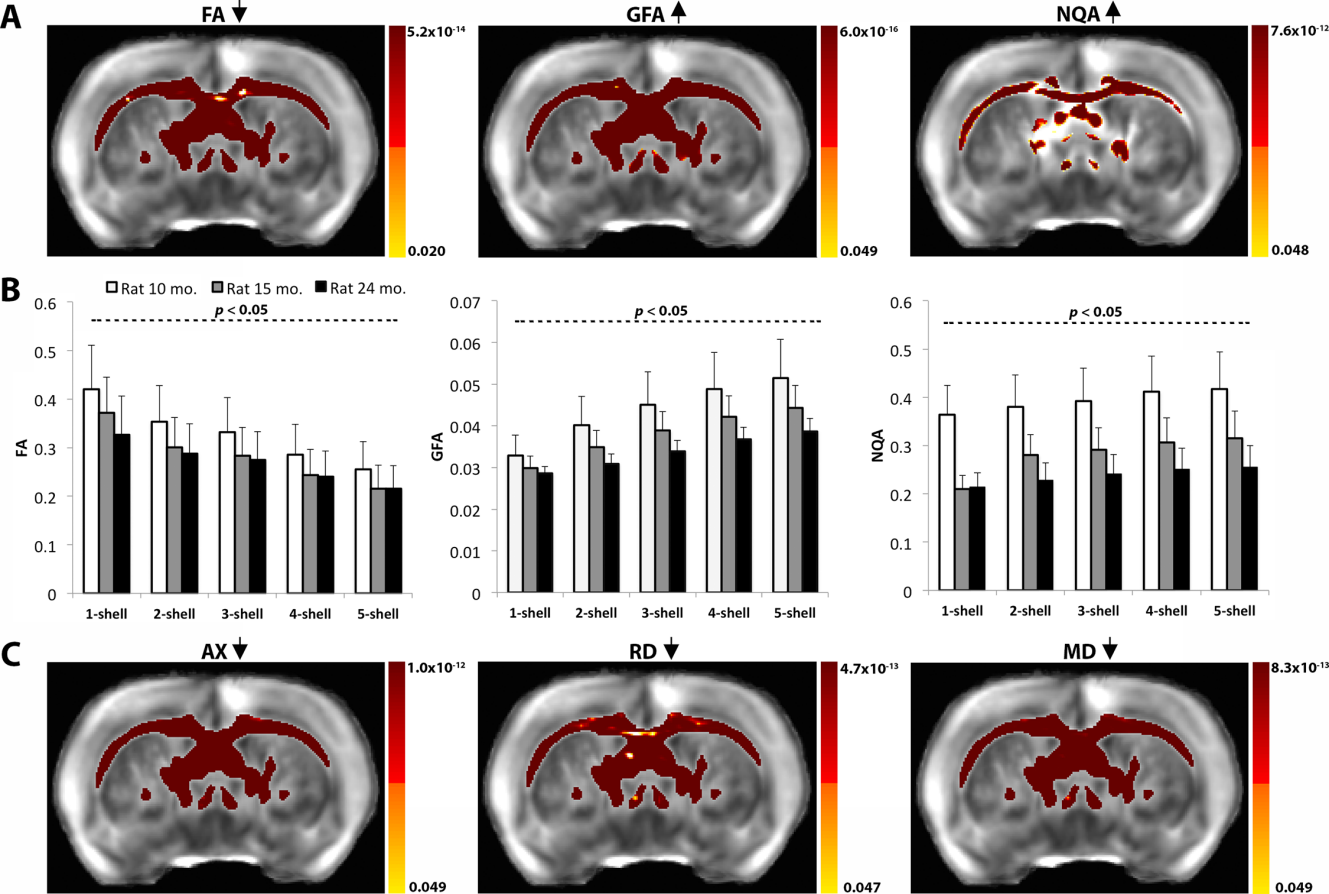
Diffusion anisotropy in the white matter changes with increasing number of *q*-sampling shells. **A.** Voxel-wise analysis results show a decrease in the apparent FA with increasing number of shells (FDR critical *P* = 0.02) and increases in QBI derived anisotropy measures, GFA and NQA, with increasing number of shells (FDR critical *P*<0.05). **B.** Mean anisotropy intensity values computed within affected white matter areas extracted from the voxel-wise analyses show decreasing (or increasing) patterns. **C.** DTI components, AX, RD and MD significantly decreased with increasing number of shells, explaining the decrease in FA from **1A**.

From our voxel-wise analyses we found that FA decreased across the white matter structure with increasing numbers of *q*-sampling shells (FDR critical *P* = 0.048) (**Fig 2A** and **2B**). The standard DTI metrics–AX, RD and MD–which are involved in computing FA, also significantly decreased with increasing number of *q*-sampling shells across the white matter (FDR critical *P*<0.05) (**Fig 2C**). On the other hand, the apparent GFA and NQA increased with increasing number of shells (FDR critical *P* = 0.049 and 0.020). We found the same significant results when we covaried for age in our statistical analyses. All three anisotropy measures were evaluated on a [0,1] scale, but note that GFA intensity values are lower than FA and NQA by a factor of 10. For this reason, direct comparisons between GFA and FA, and GFA and NQA should be performed with caution. Although not the focus of this work, it is interesting to point out the step-wise drop in anisotropy across the disease stages at 10, 15 and 24 months (**Fig 2B**). This drop in anisotropy is expected and has been previously shown in AD patients when compared to healthy controls; we will elaborate on this more in the *Discussion* section.

### Angular Deviation

**Fig 3:**
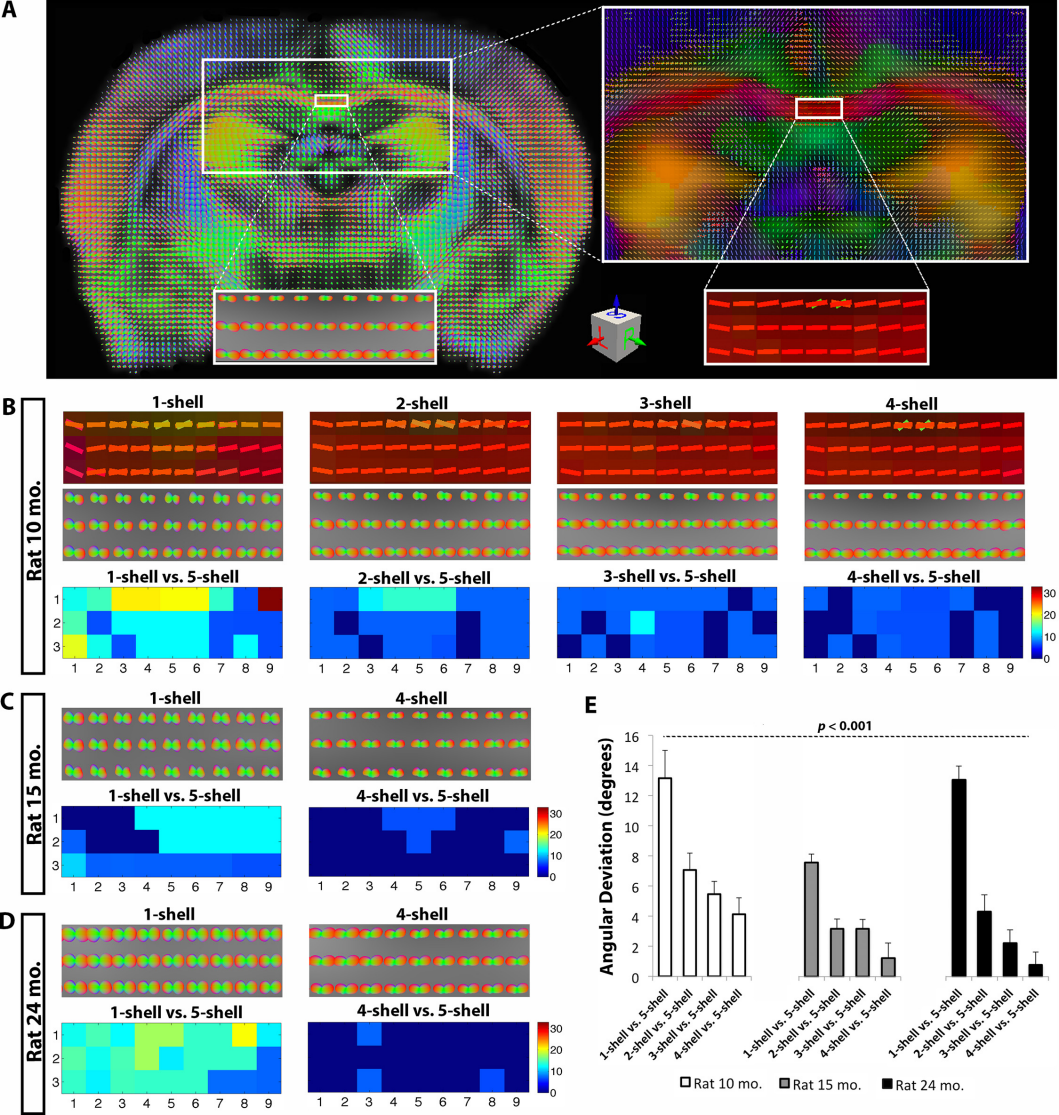
Angular deviation computed between ODF-based 1-shell, 2-shell, 3-shell and 4-shell reconstructions as compared to 5-shell reconstructions in the corpus callosum of all three rats. **A.** Coronal slice showing ODFs reconstructed with QBI and corresponding tensors in 5-shell HYDI in the 10 month old rat. **B.** ODFs and tensors in the corpus callosum and angular deviations for the 10 month old rat, **C.** 15 month old rat, and **D.** 24 month old rat. At individual voxels, angular deviations were as high as 33° between the *b* = 1000 s/mm^2^ shell and the 5-shell reconstruction. **E.** Mean angular deviation in the single- and multi-shell fibers, showing significant decrease with increasing numbers of *q*-sampling shells, compared to 5-shell HYDI (*P* = 6x10^-4^).

**Fig 4:**
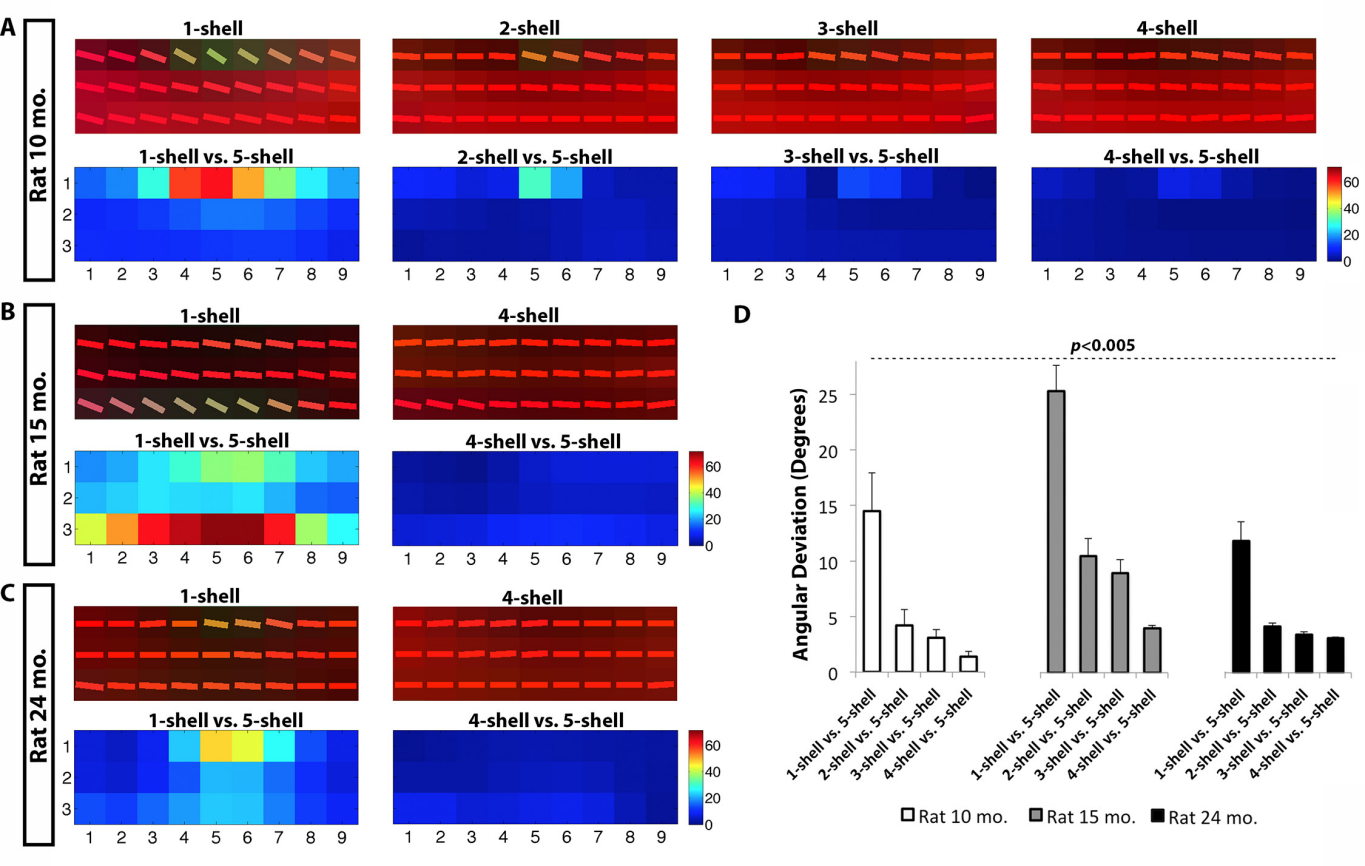
Angular deviation computed between DTI-based 1-shell, 2-shell, 3-shell and 4-shell reconstructions as compared to 5-shell reconstructions in the corpus callosum of all three rats. **B.** Tensors in the corpus callosum and angular deviations for the 10 month old rat, **C.** 15 month old rat, and **D.** 24 month old rat. Within individual voxels, angular deviations were as high as 52° between the *b* = 1000 s/mm^2^ shell and the 5-shell reconstruction. **E.** Mean angular deviation showing significant decrease of the angular deviation at each single- and multi-shell reconstruction, compared to 5-shell HYDI (*P* = 3.2x10^-3^). Note the improved fiber orientation as shells are added, correcting the inaccurate anterior-to-posterior directionality of the fibers from 1-shell (i.e., green colored tensors) to the expected left-to-right directionality (i.e., red colored tensors).

The fibers in the body of the corpus callosum are expected to run in a left-to-right direction at the midline of the brain. In other words, we expect the orientation of the tensors and ODFs to appear in a straight horizontal line (**Figs 3 and 4**), unless the voxel is so large that it catches fibers that have arched away from the midline. Both tensor-based (DTI) (**Fig 4**) and ODF-based models (QBI) (**Fig 3**) are expected to achieve proper reconstruction of fibers in this region of the corpus callosum, unlike regions with crossing fibers where the tensor based-models perform poorly. After thorough investigation, the complex 5-shell reconstructed image may best identify fiber alignment from QBI-derived ODFs (**Fig 3A**); this also applies to the tensor model where each added shell helped improve the estimates of fiber direction, leading to more accurate detection of left-to-right fibers, lowering the angular deviation when compared to the 5-shell reconstruction fiber orientation (*P* = 3.2x10^-3^). For the ODF model, deviations from the 5-shell reconstruction were as large as 33° at the worst voxel for the 1-shell reconstructed image (*b* = 1000 s/mm^2^), decreasing significantly thereafter as each *q*-sampling shell was added (*P* = 6x10^-4^). Similarly, for the DTI model, deviations from the 5-shell tensor primary orientation vectors was as high as 52° in the 1-shell, at the worst voxel, which occurred when the left-to-right fibers of the corpus callosum were confused as having anterior-to-posterior directionality (**Fig 4A** and **4B**). Note that the 4-shell reconstruction achieved comparable major fiber reconstruction to the 5-shell reconstruction schemes in both ODF- and DTI-based tensors (average angular deviation < 3°) (**Figs 3E and 4D**).

### FA-, GFA- and NQA-Aided Tractography

**Fig 5:**
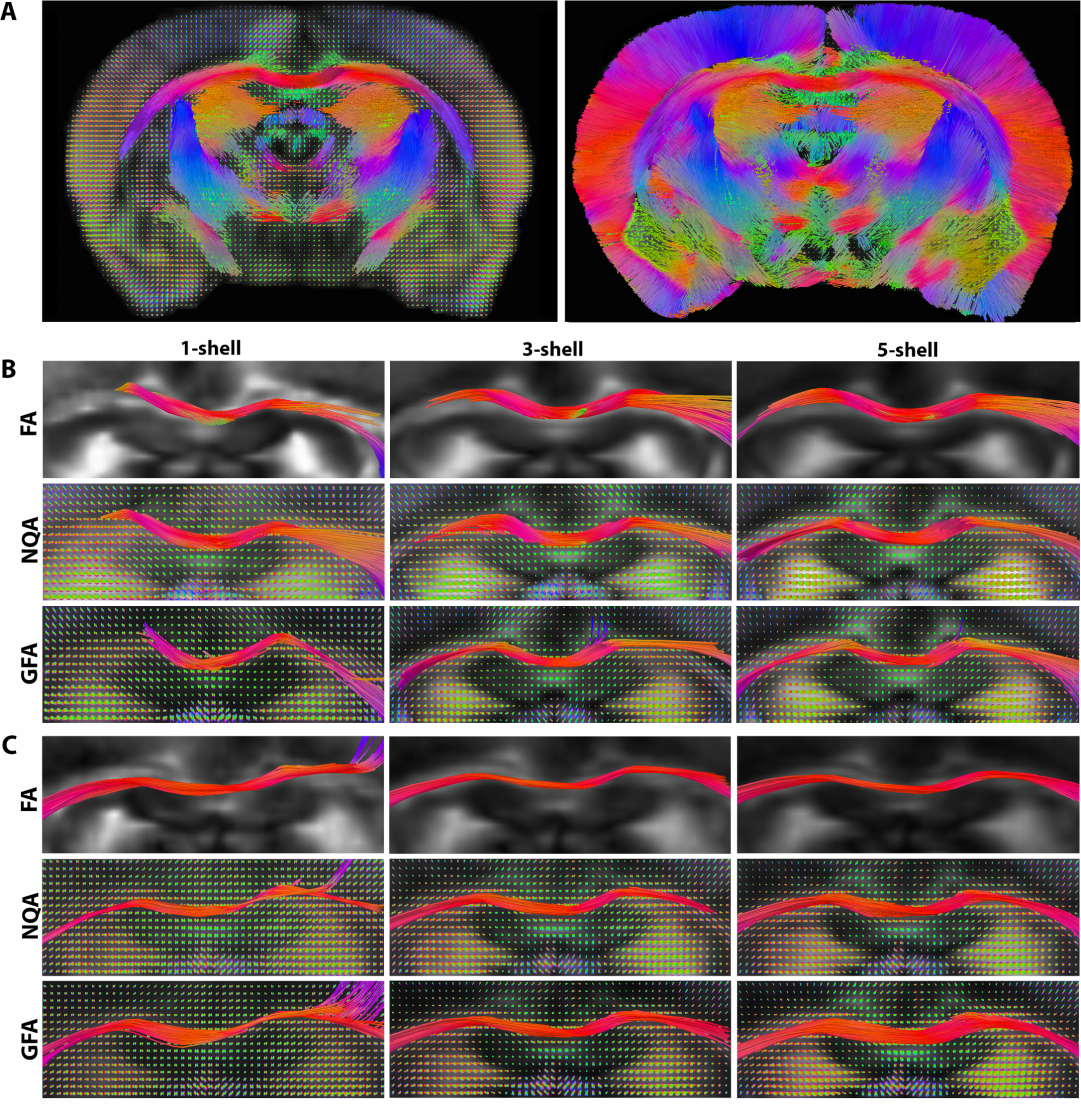
Deterministic streamline fiber tracts computed from DTI and QBI, as a function of FA, GFA and the fiber spin density NQA anisotropy values. **A.** NQA-aided tractography from QBI in the coronal slice of the 10 month old Alzheimer rat thresholded at high anisotropy values (0.25) (*left*), as well as whole coronal slice at low NQA threshold (0.10) (*right*). **B.** FA-, GFA- and NQA- aided tractography for the 10 month old Alzheimer rat and **C.** 24 month old Alzheimer rat overlaid on corresponding anisotropy maps. Note the poor resolution of some of the fiber directions in the 1-shell images versus the multi-shell images.

By studying fiber tracking performance (**Fig 5A**), we were able to better understand how angular reconstruction errors at the voxel level impact fiber reconstruction. Tracing the fibers in the corpus callosum–the section of the brain with the largest anisotropy values–is expected to be fairly straightforward, compared to more complex structures with crossing fibers. However, after observing the fibers qualitatively, we found a range of variations across the single- versus multi-shell fiber tracking outcomes, and across the selection of fiber stopping parameters.

In **Fig 5B** and **5C** we present deterministic streamline tractography in rats scanned at 10 and 24 months. Fibers obtained from the 5-shell reconstructions successfully followed the known morphology of the corpus callosum, especially from NQA-aided tractography, then GFA-aided and finally, FA-aided. Meanwhile, fibers reconstructed from 3-shell images closely followed the architecture of the fibers from the 5-shell images with slight deviations especially among the FA- and GFA-aided tracts. On the other hand, fibers traced in the 1-shell images showed abrupt stopping points although we adjusted for the fiber threshold criteria accordingly; for instance, FA values in the 1-shell images were higher compared to multi-shell FA, as seen in our voxel-wise analyses, so we used a 0.25 threshold (versus 0.2 in multi-shell). On the other hand, since NQA and GFA intensity values were lower in the 1-shell image than in the multi-shell images, we adjusted the thresholds accordingly to 0.25 and 0.02 (versus 0.3 and 0.03 in multi-shell). Another observation among the 1-shell reconstructed fibers was incoherent tracts running between the corpus callosum and gray matter structure in the 24 month old Alzheimer rat. Also, fibers that may appear to have stopped abruptly in the corpus callosum in the 1-shell, and oftentimes 3-shell images, continued in the adjacent voxels not visible in the consistent coronal slices we used to illustrate all the findings in **Fig 5**. To explain further, the route of the same fiber bundle in the coronal slice in the 5-shell reconstruction was more inconsistent when traced in the 1-shell images–where segments of the same fiber bundle were reconstructed across multiple coronal slices, or a different set of voxels.

### Changes in Apparent Anisotropy in the Hippocampus

**Fig 6:**
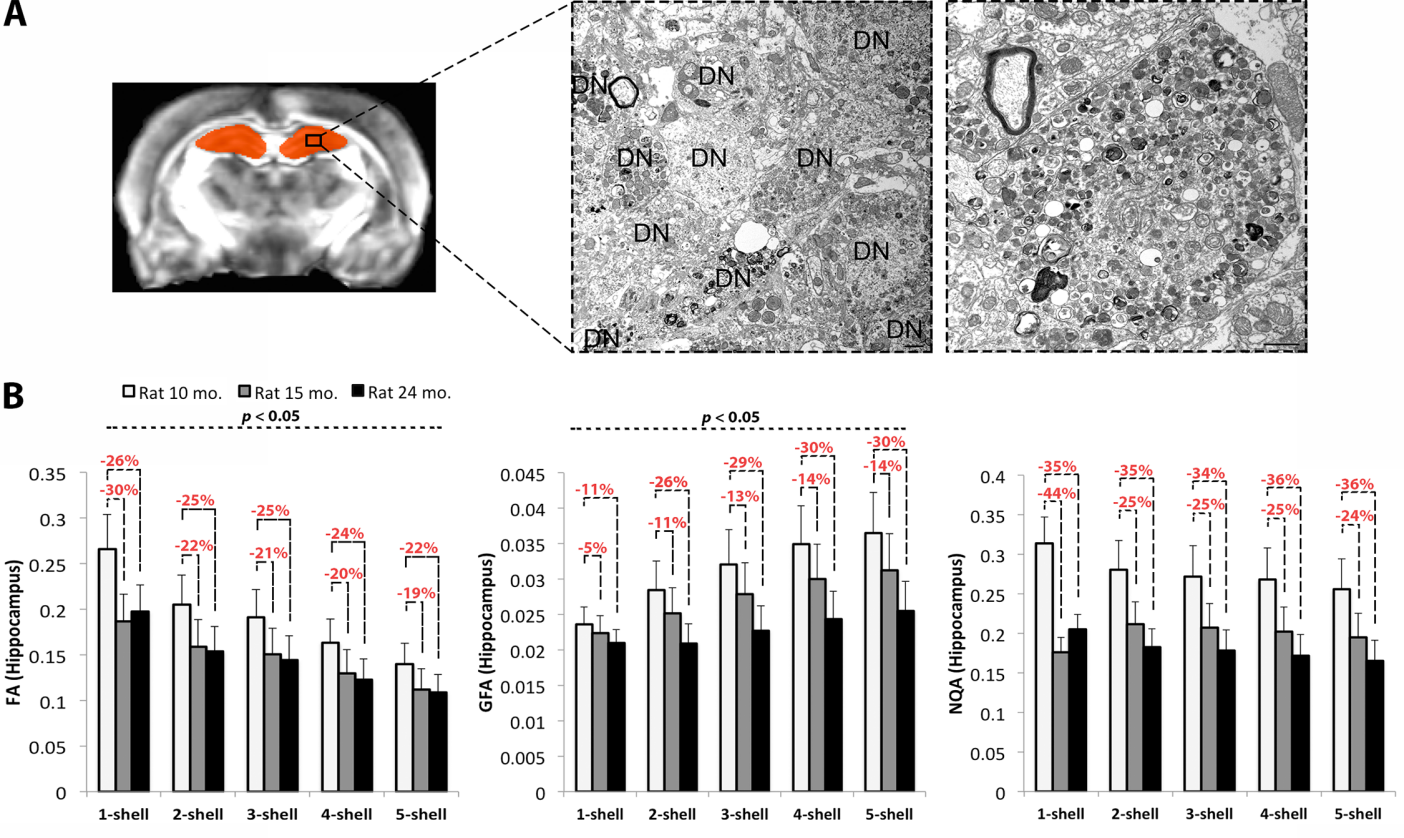
Gray matter alterations in the hippocampus and diffusion anisotropy with HYDI. **A.** Transmission electron micrographs from 16 month old TgF344-AD rat hippocampus show numerous dystrophic neurites (DN, *left panel*), which are swollen at higher magnification (*right panel*). Scale bars denote 500 nm. **B**. Diffusion anisotropy in the hippocampus with decreases in the apparent FA (*P* = 6.6x10^-5^) and increases in GFA (*P* = 1.8x10^-4^) with increasing number of *q*-sampling shells. NQA anisotropy did not change significantly with the number of shells, but it was most indicative of the expected step-wise drop in diffusion anisotropy across the different time points. Note slight inconsistencies for FA and NQA at 1-shell; multi-shell reconstructions may allow more reliable estimations.

Mean FA in the hippocampus decreased with increasing number of shells (*P* = 6.6x10^-5^), while mean GFA increased with the number of shells (*P* = 1.8x10^-4^) (**Fig 6**). These results were robust even when we covaried for age. They were also consistent with the anisotropy patterns detected in the corpus callosum. Mean NQA did not change significantly in the hippocampus across the multi-shell scheme. For each measure, we observed a consistent step-wise drop in diffusion anisotropy across the three time points in all measures computed with the multi-shell images; there were some inconsistencies among the decreases in anisotropy detected by FA and NQA for the 1-shell images–with larger drops detected at 15 months than at 24 months. As these inconsistencies were found only for the 1-shell images, they might indicate that the multi-shell images provide more reliable estimations of diffusion anisotropy in the gray matter of the AD rat.

## Discussion

In this paper, we apply a high-field multi-shell acquisition technique to explore and visualize white and gray matter properties, from standard measures used widely in clinical neuroradiology to more complex high-order metrics. In explaining the technique, we illustrated the benefits of HYDI, with experiments that show how each *q*-sampling shell adds to the complexity of the reconstructed diffusion signal. For the first time, we demonstrate microstructural properties from MRI in TgF344-AD transgenic Alzheimer rats. To do this, we scanned rats with 300 diffusion volumes and a wide range of *b*-values–*b* = 1000, 3000, 4000, 8000 and 12000 (s/mm^2^). We investigated the commonly studied FA maps across single- and multi-shell reconstructions, in addition to more complex anisotropy maps, GFA–obtained from the ODF function and NQA–computed from the fiber spin density from QBI. To our knowledge, this is the first study to demonstrate voxel-wise differences in these anisotropy maps across various multi-shell schemes. Furthermore, we studied the angular discrepancies of reconstructed tensors and diffusion ODFs in a region with known anatomical white matter morphology and performed FA-, GFA- and NQA-aided tractography to elucidate the effects of these angular deviations. We discuss our key findings below.

As we add higher *b*-value shells, there are significant changes in the intensity of the tensor- and ODF-based anisotropy maps. The powerful gradient strength and long diffusion times allow higher *b*-value shells, complete dephasing and signal loss for fast moving water molecules along the axons [33]. They also lead to loss of highly anisotropic signal in the intra-axonal compartment, so there is a known drop in FA with the addition of each *q*-sampling in the multi-shell reconstruction scheme (**Fig 2**). This phenomenon is further supported by the simultaneous decrease in other standard DTI tensor metrics, AX, MD and RD, as each shell is added. Each *q*-sampling shell had a higher *b*-value leading to more signal loss. Typically, the SNR is highest in the low *b-*value shell (**Fig 1A**) while the CNR is highest in the high *b*-value shells (**Fig 1C**) [11] [16]. As we added more *q*-sampling shells, the SNR dropped (**Fig 1A**)–also shown in our previous work [34], leading to a decrease in the magnitude of all DTI metrics. Meanwhile, the higher *b*-value shells led to “sharper” angular diffusion profiles that are more sensitive to fiber orientation [35] [33]. This can benefit the QBI derived metrics GFA and NQA: these increased as each higher *b*-value shell was added. This is expected as the ODF profile becomes more ‘concentrated’ [10] and complex with addition of each shell.

For applications such as white matter tractography, we found that multi-shell reconstructions improved the recovery of major fiber orientations in both tensor- and ODF-based reconstructions. Added higher *b*-value shells seemed to improve the tensor and ODF estimations leading to fewer artifactual fibers. The angular deviation between tensor- and ODF-based 2- and 3-shell HYDI versus 5-shell HYDI were on average < 6° in the body of the corpus callosum, possibly indicating that 2- and 3-shell HYDI can perform comparably well as 5-shell HYDI (**Figs 3 and 4**), while also saving scanning time. Prior studies have shown that in a HARDI reconstruction scheme (with *b*-values shells evaluated separately, not in a HYDI setup), increasing the *b*-value from 1000 s/mm^2^ to 3000 s/mm^2^ can reduce the minimal resolvable angle between tracts from approximately 45° to 30°; however, raising the *b*-value to 5000 s/mm^2^ did not show any further significant improvements independent of the number of diffusion encoding gradients [35] [33]. These findings were based on an ODF reconstruction (specifically a deconvoluted diffusion ODF, or a fiber ODF) and were independent of the number of degrees of freedom (i.e. spherical harmonic order) [35]. Even so, in this work we found that single-shell diffusion ODF reconstructions led to angular deviations > 13° (averaged across the studied section of the corpus callosum) and as high as 33° compared to 5-shell HYDI within individual voxels (**Fig 3**), causing changes in the directionality of fibers from their expected left-to-right orientation (**Fig 5**). These deviations were more pronounced in the single-shell DTI-based reconstructions (angular deviation > 17°) and as high as 52° at individual voxels when compared to 5-shell HYDI DTI tensor directionalities (**Fig 4**). Also, changes in anisotropy level and angular deviation in the corpus callosum with increasing number of shells were found to be independent of the aging effect in these animals. In this study, we focused on homogeneous fibers that can be, in theory, successfully reconstructed with both DTI and QBI methods and allow us to understand the deviations between single- and multi-shell reconstruction schemes without the added complexity of crossing fibers and various reconstructions. However, future studies are needed to investigate the characterization of complex tissue organization and application of other ODF estimation methods (i.e., spherical deconvolution [36], or tensor distribution functions).

Overall, studies employing HYDI imaging techniques are not as common as studies using HARDI and DWI. Sampling the entire *q*-space can lead to long scan times that tend to increase patient attrition. Therefore, despite its great utility, HYDI is infrequently used in clinical applications. By contrast, in neuroradiology research–such as connectomics–HYDI and *q*-space imaging are popular. Both DTI and HARDI have been proposed as sources of neuroimaging biomarkers for clinical trials and longitudinal monitoring, in traumatic brain injury, for example. With HYDI, lesions may become more apparent and easier to identify due to the high CNR, otherwise not obtainable in DTI and HARDI. In addition, highly detailed microstructural maps can be estimated with a multi-shell scheme, such as the computation of axonal diameter maps [37] [38] that reveal the diffusion signal within different compartments of the white matter, reflecting intra- and extra-axonal tissue properties. These can offer invaluable data on white matter tissue characteristics that might be informative of excessive intra- or extra-axonal volume, dystrophic neurites, or even neuroinflammation. These mechanisms are often reported as a key component in the etiology of neurodegenerative diseases such as AD [39], and are not fully captured by commonly used DTI measures or even by HARDI ODF-derived metrics. Clearly, correlative work is needed to verify the cellular correlates of all these signals (36) and the biological processes that affect them.

### Trade-offs and Pragmatic Considerations

To develop efficient measurement strategies for HYDI in a clinically feasible scan time, one may consider reducing the number of outer shells or limit the angular resolution in the lower *b*-value shells. In this study, we used a fixed number of encoding directions (60 diffusion volumes/shell in each of the 5 shells) and showed that 3-shell HYDI performs comparably well to 5-shell HYDI. Very high *b*-value shells (i.e., *b* = 8000 or 12000 s/mm^2^) might not provide significant improvement in interpreting the diffusion signal (also suggested by [35]). Exclusion of higher *b*-values could save scan time and maintain a relatively high SNR for the primary white matter metrics of interest, including tractography. The SNR in the *b* = 1000 s/mm^2^ DWI shell was highest (44) and decreased thereafter (i.e., SNR at *b* = 12000 s/mm^2^ was 17), but when the multi-shell SNR was averaged across the individual shells, it was higher than in most individual shells (SNR at 5-shell was 28) (**Fig 1**). Note that the SNR will differ in human DWIs from animal scans, but similar mathematical principles apply. Some studies suggest that SNR in the DWIs > 10 is sufficient to resolve the peaks in the fiber orientation density [33] [35] but this requirement should be assessed in more detail. Another advantage of reducing the number of outer shells is limiting the long TE–often leading to suboptimal levels of noise [8] [11] that can also lower the SNR in the lower shells. Moreover, another option to make HYDI clinically feasible is to increase the angular resolution with the level of diffusion weighting as described by Wu and colleagues, who used as few as 3 DWI volumes in the lowest shell (*b* = 375 s/mm^2^) and 50 DWI volumes in the 5^th^ outer shell (*b* = 9375 s/mm^2^). To benefit most from their data, they performed DTI on the most inner shells and QBI on the outer shells [11].

Although not the focus of our study, it is important to consider that anisotropy measures can be affected with disease progression or aging, which is what makes them valuable neuroradiologic markers (note the decreased anisotropy values across the transgenic Alzheimer rats at the three time points in **Fig 2B**). We have previously shown that this specific TgF344-AD rat model presents with dystrophic neurites in the hippocampus (**Fig 6A**) and cortex [18]. The dystrophic neurites were characterized by enlarged axons and dendrites as they got filled with vacuoles over time [40]. While we do not have healthy rats to statistically test for changes with disease progression–which is a major limitation of this study–we used our younger rat (10 month old) as a control subject to compare against suggestive changes at the later time points (**Figs 2B and 6**). Given that the corpus callosum has not been studied in this rat model, we instead examined the hippocampus. Although gray matter deficiencies may not directly translate to white matter alterations, the pathological findings in the gray matter structures may lead to the secondary white matter alterations as we detected here. Multi-shell images may better estimate changes in diffusion anisotropy in the hippocampus of the transgenic rats, whereas 1-shell reconstructions might lead to inconsistencies. Anisotropy levels are expected to drop with AD in both the white and gray matter, as barriers to water diffusion created by the neuronal structures are damaged. All three measures obtained from multi-shell images indicated a step-wise drop in anisotropy with disease progression best quantified by NQA (Fig 6B). This may be linked with age-dependent neuronal loss and neurodegeneration previously found in the hippocampus of these rats [18].

Another limitation is the low number of experimental subjects that also prevents us from statistically differentiating between the disease stages of the transgenic Alzheimer rat model. However, the suggested pattern of decreased anisotropy has been previously shown in clinical studies of AD patients [41]. Nonetheless, due to known drops in anisotropy in patients with dementia, it is important to accordingly adjust fiber tracing stopping criteria that depend on anisotropy values. Here, we ran streamline deterministic tractography (**Fig 5**) and lowered the anisotropy termination criterion in the 24 month old rats to be able to track the fibers in the corpus callosum. We also used lower stopping anisotropy thresholds for the fibers traced in single-shell versus the multi-shell HYDI across all anisotropy measures. Many fiber tracking methods still use voxel-based approaches to determine when the fibers should be terminated, but these approaches cannot selectively remove noisy fibers as all fiber directionalities within a voxel share the same anisotropy values [32]. Therefore, voxel-based indexes such as FA and GFA will show susceptibility for partial volume effects and might terminate early or continue inaccurately. Unlike FA and GFA, NQA is an ODF-based index computed from the peak orientation of each ODF and is defined for each fiber orientation [3]. NQA-aided tractography might better facilitate fiber tracking studies, also shown in [3].

In this paper we show a basic demonstration of HYDI in transgenic Alzheimer rats. To our knowledge, this is the first study to acquire 300 DWIs across 5 distinct *q*-sampling shells to demonstrate and visualize how a range of standard and advanced metrics differ in single- versus multi-shell scenarios, as well as tractography. We find that HYDI significantly improves fiber reconstruction for both ODF- and tensor-based models leading to more accurate fiber tracking in the white matter. Scan time for 5-shell HYDI might not yet be feasible in a clinical setting, but the added value of 2 or even 1 extra shell could boost the accuracy of the diffusion signal and aid the outcomes of clinical research and especially, more advanced human connectomics projects to map the brain’s neural pathways and networks.
